# Improving prediction of phenotypic drug response on cancer cell lines using deep convolutional network

**DOI:** 10.1186/s12859-019-2910-6

**Published:** 2019-07-29

**Authors:** Pengfei Liu, Hongjian Li, Shuai Li, Kwong-Sak Leung

**Affiliations:** 1 Department of Computer Science and Engineering, the Chinese University of Hong Kong, Sha Tin, N.T., Hong Kong, China; 2 SDIVF R&D Centre, Hong Kong Science Park, Sha Tin, N.T., Hong Kong, China; 3 CUHK-SDU Reproductive Genetics Joint Laboratory, School of Biomedical Sciences, the Chinese University of Hong Kong, Sha Tin, N.T., Hong Kong, China

**Keywords:** Phenotypic screening, Deep learning, Convolutional network, GDSC

## Abstract

**Background:**

Understanding the phenotypic drug response on cancer cell lines plays a vital role in anti-cancer drug discovery and re-purposing. The Genomics of Drug Sensitivity in Cancer (GDSC) database provides open data for researchers in phenotypic screening to build and test their models. Previously, most research in these areas starts from the molecular fingerprints or physiochemical features of drugs, instead of their structures.

**Results:**

In this paper, a model called twin Convolutional Neural Network for drugs in SMILES format (tCNNS) is introduced for phenotypic screening. tCNNS uses a convolutional network to extract features for drugs from their simplified molecular input line entry specification (SMILES) format and uses another convolutional network to extract features for cancer cell lines from the genetic feature vectors respectively. After that, a fully connected network is used to predict the interaction between the drugs and the cancer cell lines. When the training set and the testing set are divided based on the interaction pairs between drugs and cell lines, tCNNS achieves 0.826, 0.831 for the mean and top quartile of the coefficient of determinant (*R*^2^) respectively and 0.909, 0.912 for the mean and top quartile of the Pearson correlation (*R*_*p*_) respectively, which are significantly better than those of the previous works (Ammad-Ud-Din et al., J Chem Inf Model 54:2347–9, 2014), (Haider et al., PLoS ONE 10:0144490, 2015), (Menden et al., PLoS ONE 8:61318, 2013). However, when the training set and the testing set are divided exclusively based on drugs or cell lines, the performance of tCNNS decreases significantly and *R*_*p*_ and *R*^2^ drop to barely above 0.

**Conclusions:**

Our approach is able to predict the drug effects on cancer cell lines with high accuracy, and its performance remains stable with less but high-quality data, and with fewer features for the cancer cell lines. tCNNS can also solve the problem of outliers in other feature space. Besides achieving high scores in these statistical metrics, tCNNS also provides some insights into the phenotypic screening. However, the performance of tCNNS drops in the blind test.

**Electronic supplementary material:**

The online version of this article (10.1186/s12859-019-2910-6) contains supplementary material, which is available to authorized users.

## Background

Historically, drug discovery was phenotypic by nature. Small organic molecules exhibiting observable phenotypic activity (e.g. whole-cell activity) were detected, a famous example being penicillin, which was serendipitously found. Phenotypic screening, an original drug screening paradigm, is now gaining new attention given the fact that in recent years the number of approved drugs discovered through phenotypic screens has exceeded those discovered through molecular target-based approaches. The latter, despite being the main drug discovery paradigm in the past 25 years, can potentially suffer from the failure in identifying and validating the therapeutic targets. In reality, most FDA approvals of first-in-class drugs actually originated from phenotypic screening before their precise mechanisms of actions or molecular targets were elaborated. A popular example of this is aspirin (acetylsalicylic acid), for which it took nearly a century to elucidate the mechanism of its actions and molecular targets.

There are some public phenotypic screening datasets online to support the study of the pharmacological functions of drugs. Cancer Cell Line Encyclopedia (CCLE) and Genomics of Drug Sensitivity in Cancer (GDSC) are the most popular datasets in the field [1].

A pioneer work using machine-learning approaches to predict drug response on cancer cell lines was by Menden et al. [2]. The authors used a neural network to analyze the response of drugs to cancer cell lines on the GDSC dataset. Their main result was the achievement of 0.72 for the coefficient of determination and 0.85 for the Pearson correlation. [3] and [4] are two other works on GDSC dataset. The first one used kernelized Bayesian matrix factorization to conduct QSAR analysis on cancer cell lines and anti-cancer drugs, and the second one used multivariate random forests. Both of their results were not as good as those in [2], which is chosen to be the baseline for our work.

The first wave of applications of deep learning in pharmaceutical research has emerged in recent years. Its utility has gone beyond bioactivity predictions and has shown promise in addressing diverse problems in drug discovery. Examples cover bioactivity prediction [5], de novo molecular design [6], synthesis prediction [7] and biological image analysis [8, 9]. A typical example of applying deep learning in protein-ligand interaction prediction is the investigation done by Ragoza et al. [10].

Convolutional neural network (CNN) is a machine learning model that can detect relevant patterns in data and support classification and regression [11]. CNN has achieved breaking-through results in many areas, including pharmaceutical research [12–14] and has won the championship in ImageNet-2012 [15].

Inspired by the achievements of CNN in these areas, we are interested to see if CNN, compared to conventional machine-learning techniques [2–4], could significantly improve the prediction accuracy of phenotypic drug response on cancer cell lines. In this paper, a twin CNN networks model called tCNNS is introduced to predict the drug cell line interaction. tCNNS comprises a CNN for drugs and another CNN for cancer cell lines, which will be explained in detail later. The latest version of the GDSC dataset is adopted to evaluate the performance of tCNNS. Unlike previous works, here the structure of tCNNS is advanced, and it is tested on the bigger and more complete dataset. Most importantly, it achieves much better results than previous works. We share our model online, hoping to make a contribution to other researchers.

## Related work

Erik et al. [16] stated that both the qualitative classifiers and the quantitative structure-activity relationship (QSAR) models in drug discovery depend on the molecular descriptors, which is *the decisive step in the model development process*.

Recently, in drug discovery, researchers started to use the molecular structure of drugs directly as features [17–20] instead of using extracted features from open source software [21, 22]. Due to their good ability to process high-dimensional structure data, deep learning has been largely adopted in this area [16, 23, 24].

From the perspective of machine learning, drug cell line interaction analysis can be considered as a classification task where the outputs are some categorical values, such as sensitivity or resistance, or a regression task where the outputs are some numerical values, such as IC_50_. Wang et al. [25] used support vector machine (SVM) to handle the classification problem by merging drug features from different sources, such as the chemical properties and the protein targets. The features they used to represent cell lines are the same as ours, which are the copy number variations, gene mutation states and expressions. Rahman et al. [26] built a random forest based ensemble model for drug sensitivity prediction and they found that the information of cancer types can help researchers to enhance the performance even with a fewer number of samples for training. Ding et al. [27] used the elastic net to generate a logistic model to predict drug sensitivity. Zhang et al. [28] applied another approach on the classification problem. It predicted interaction labels using a drug-drug similarity network and a cell line-cell line similarity network. These similarity networks were computed based on the features of drugs and cell lines respectively.

Regression is more challenging than classification because there are infinite possible outputs, and many machine learning models have been adopted to handle it. Among them, matrix factorization (MF) and neural network (NN) are the two most widely used models and have been proven to be most useful. In MF, the drug target interaction matrix is decomposed into two low-rank matrices, and the interactions among drugs and targets are represented by the inner products of the vectors in the two low-rank matrices. Ammad et al. [3, 29] designed a kernelized Bayesian matrix factorization method for drug cell line interaction prediction and reported their *R*^2^ based on GDSC, which are not as good as the results in Menden et al. [2]. Chayaporn et al. [30] modified an MF based recommendation system algorithm and applied it to drug cell line interaction. The authors tested their algorithms on GDSC and reported the Spearman correlation as 0.6. Alexander et al. [31] came up with a deep neural network to predict the pharmacological properties of drugs and drug repurposing. They built a fully connected network and the input features for drugs were the gene level transcriptomic data, which were processed using a pathway activation scoring algorithm.

Simplified molecular input line entry specification (SMILES) of the drugs is converted into vectors using unsupervised auto-encoder [17, 32]. These vectors can be used as features or fingerprints of drugs. This method was further extended for drug discovery by Han et al. [20] and Zheng et al. [33]. The authors predicted the use of drugs by comparing the similarity between those vectors of drugs.

In the recent two years, there are several different deep neural network (DNN) models that were trained directly from drug structures and avoided the *decisive step*. These DNN models include unsupervised auto-encoder (AE), supervised convolution neural network(CNN), and recurrent neural network (RNN).

Although it is attractive to apply CNN to the formulas of drugs, it is also very difficult to do so because there is no uniform pattern in the drug formulas. Instead, researchers tried to apply CNN on the image of the formulas of drugs as an alternative solution. Goh et al. [34] adopted a computer vision method to screen the image of drugs. The advantage of starting from the image of drugs rather than from their formulas is that it can avoid the massive work of handling the diversity of drugs. However, the disadvantages are that the accuracy is compromised because the information will be distorted when mapping drug structures to images and the performance of this method relies on the quality of the image processing.

Beyond the application of applying CNN to drug images, it is also possible to apply CNN to molecular 3D structures directly. Wallach et al. [35] predicted the binding energy of the small area around an atom, rather than on the entire structure of drugs. It is interesting to compare the different representations of drugs, such as the 3D structured, the feature vectors learned from SMILES and the features extracted from other software like PaDEL [36]. They may have different influences on different problems.

Even though RNN is usually used to handle time sequence data [37] instead of spatial data, it is very impressive that Lusci et al. [38] applied RNN to the SMILES of drugs to predict their solubility. The authors converted the SMILES into indirect graphs, and then fed them into an RNN. In their work, the authors only considered the property of drugs alone, without considering the interactions among drugs and other biological factors, such as cell lines or proteins.

We compare our model to that by Menden et al. [2], where the authors used a neural network to analyze the IC_50_ of drugs to cancer cells on the same dataset as ours. However, their network structure is not advanced enough, and the features they used are not informative enough. We designed tCNNS, a convolution neural network (CNN) based model, to predict the interaction between drugs and cell lines.

## Methods

In this section, the chosen database GDSC, the preprocessing steps, and the proposed neural network structure are described in detail to make our experiments easier to replicate.

### Data acquisition and preprocessing

Genomics of Drugs Sensitivity in Cancer (GDSC) [39] is a public online database about the relationship among many types of cancer and various anti-cancer drugs. Cancer cell lines in GDSC are described by their genetic features, such as mutations state and copy number variances. For the drugs, GDSC provides their names and the compound id (CID). In chemistry, CID is a unique number assigned to each molecule and can be used as the reference number to extract more information about the drugs such as their molecular structures from other databases. GDSC uses IC_50_ as the metric of drugs’ effectiveness on cancers. IC_50_ is the amount of drug needed to inhibit a cancer by half. The less the value is, the more effective the drug is. GDSC is an ongoing project and is being updated regularly. In our paper, GDSC version 6.0 is used. As a comparison, Menden et al. [2] used version 2.0 of the GDSC, which contains much fewer drugs and cell lines.

The three downloaded files from GDSC are:

- Drug _list.csv, which is a list of 265 drugs. Each drug can be referred to by its CID or name.
- PANCANCER _*Genetic*_feature.csv, which is a list of 990 cancer cell lines from 23 different types of cancers. Each cell line is described by at most 735 features. Any feature belongs to one of the two categories: mutation state or copy number alteration.
- PANCANCER _IC.csv, which contains the IC_50_ information between 250 drugs and 1074 cell lines.

Note that the numbers of drugs in files (a) and (c) are inconsistent, and that the numbers of cell lines in files (b) and (c) are also inconsistent. Some cell lines have less than 735 features. Besides, GDSC does not provide the features for drugs, which have to be downloaded from other datasets. All of these indicate that three preprocessing steps are needed to clean the data.

1. The first step is to cleanse the drug list. There 15 repeating items in file (a), which are removed. Some CIDs in file (a) are inconsistent with the CIDs found in PubChem [40], which is a popular public chemical compounds database. To enforce the consistency, the CIDs from PubChem have been adopted. Some drugs cannot be found in PubChem by referring to their names in the file (a) and they are removed. As a result, 223 drugs with both names and CIDs are left.
2. The second step is to cleanse the cell lines list. For the 990 cell lines in file (b), 42 of them has less than 735 features. After the removal, 948 cell lines are left.
3. In the third step, only the IC_50_ values between the remaining drugs after the first step and the remaining cell lines after the second step are used. All the other IC_50_ values in file (c) are removed. In summary, there are 223 drugs and 948 cell lines after preprocessing. Among the 223×948=211,404 interacting pairs, 81.4*%* (172,114) of the IC_50_ values are provided in file (c), whereas 18.6*%* (39,290) are missing, which are also taken out.

The IC_50_ data in file (c) are the logarithm of their real value. To make it easy for training and comparison, the method reported in [2] is used to normalize the logarithmic IC_50_ values in the (0,1) interval. Given a logarithmic IC_50_ value *x*, the real value *y*=*e*^*x*^ is got by taking the exponential formal of *x*, and the following function is used to normalize *y*:

$$y \mapsto \frac{1}{1 + y^{-0.1}}\,. $$

Usually *y* is very small (< 10^−3^), and the parameter value − 0.1 has been chosen to distribute the result more uniformly on the interval (0,1) [2].

### Numerical descriptor extraction

Recently, there are some pioneering works that apply deep neural network (DNN) directly to the simplified molecular-input line-entry system (SMILES) of drugs. SMILES is a linear notation form to represent the structure of molecules, in which letters, digits and special characters are used to represent the chemical elements in a molecule. For example, “C” stands for carbon atom and “=” is for covalent bond between two atoms. Carbon dioxide can be represent as O=C=O and aspirin can be represented as O=C(C)OC1CCCCC1C(=O)O.

There are some challenges to apply CNN on drugs in SMILES format: first, SMILES can be constructed in various ways and there can be many possible SMILESs for each drug; second, the size of the samples for a CNN should be consistent, but the lengths of the SMILES format of drugs are different from each other; third, and more importantly, the SMILES descriptions are composed of different letters representing different chemical elements, such as atoms and bonds, and it does not make sense to apply convolution operation among different chemical elements. To solve these problems, preprocessing is needed to convert the SMILES into a uniform format, so that different chemical elements are separated from each other and are independently treated under CNN.

To keep unique SMILES format for the drugs, the *canonical SMILES* [41] is adopted as the representation for the drugs. Among 223 drugs, 184 canonical SMILES have been found from PubChem by the drug names, using a python interface for PubChem. The canonical SMILES of the remaining 39 drugs are downloaded from the Library of the Integrated Network-based Cellular Signatures (LINCS) [42].

The longest SMILES for the drugs contains 188 symbols, and most SMILES lengths are between 20 and 90. To keep the size consistent and retain the complete information, all SMILESs are left aligned with space padding on the right if they are shorter than 188.

The neural network cannot directly take the drugs in SMILES format as input, and it is needed to convert the SMILES format (they are of uniform length now after handling the second challenge) into a format that can be used in the neural network. There are 72 different symbols in the SMILES format for the total 223 drugs. The distribution of these symbols is quite unbalanced. For example, carbon atom [C] appears in all the 223 drugs. Meanwhile, there is only one drug containing [Au] and only one drug containing [Cl]. Suppose the rows are used to represent different symbols, and the columns are used to represent positions in the SMILES format, then each drug in SMILES format can be converted into a 72∗188 one-hot matrix which only contains 0 and 1. In the one-hot matrix for a drug, a value 1 at row *i* and column *j* means that the *i*th symbol appears at *j*th position in the SMILES format for the drug. In tCNNS, each row of the one-hot matrix is treated as a different channel in CNN, and the 1D convolutional operation will be applied along each row of the one-hot matrix, which restricts convolutional operation within the same chemical element.

### Deep neural network

**Fig. 1:**
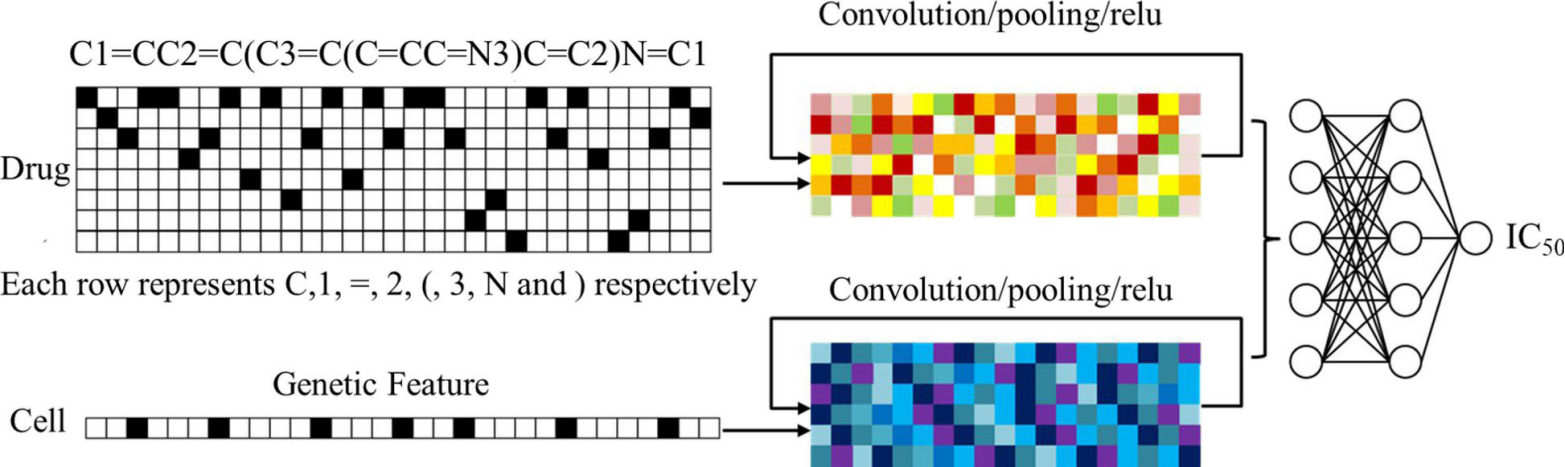
The upper part is the branch for drugs, and the lower part is the branch for cell lines. Both are inputs of a fully connected network on the right-hand side. The general work-flow of our model is from left to right. The left-hand side is the input data of one-hot representations for drugs and the feature vectors for cell lines. The black square stands for 1 and empty square stands for 0. In the middle, there are a CNN branch to process the drug inputs and a CNN branch to process cell lines inputs respectively. They take the one-hot representations and feature vectors as input data respectively, and their outputs can be interpreted as the abstract features for drugs and cell lines. The structures of the two convolution neural networks are similar. The right-hand side is a fully connected network that does regression analysis from the IC_50_ to the abstract features from the two CNNs in the middle part

The structure of the proposed model tCNNS is shown in Fig. 1. Its input data consist of the one-hot representation of drugs (phenanthroline is used as an example for the drugs) and the feature vectors of the cell lines. The work-flow can be divided into two stages as follows.

First stage: A model with two CNN branches is built to distil features for drugs and cell lines separately. A 1D CNN is used for the cell-line branch since the input data are 1D feature vectors for cell lines. Another 1D CNN is used for the drug branch and treat different symbols as different channels in the CNN. The convolution is applied along the length of the SMILES format. The structures for the two branches are the same. For each branch, there are three similar layers: each layer with convolution width 7, convolution stride 1, max pooling width 3, and pooling stride 3. The only difference between the layers is that their number of channels are 40, 80 and 60, respectively. The choices of these parameters for the CNN are inspired by the model in [43], in which the author chose a three-layers network model and used a prime number as filter width. It is found that either reducing the pooling size or adding the channel number has the potential to enhance the proposed model but with the cost of losing stability. Losing stability means that experimental results sometimes become unrepeatable. This problem will be detailed in “Results” section.

Second stage: After the two branches of the CNN, there is a fully connected network (FCN), which aims to do the regression analysis between the output of the two branches and the IC_50_ values. There are three hidden layers in the FCN, each with 1024 neurons. The dropout probability is set to be 0.5 for the FCN during the training phase [43].

tCNNS is implemented using TensorFlow v1.4.0 [44], which is a popular DNN library with many successful applications [44, 45].

### Performance measures

Three metrics are adopted to measure the performance of our model: the coefficient of determination (*R*^2^), Pearson correlation coefficient (*R*_*p*_), and root mean square error (RMSE). This is the same as that in the benchmark paper [2].

*R*^2^ measures variance proportion of the dependent variables that is predictable from the independent variables. Let *y*_*i*_ be the label of a sample *x*_*i*_, and our label prediction on *x*_*i*_ is *f*_*i*_. The error of our prediction, or residual, is defined as *e*_*i*_=*y*_*i*_−*f*_*i*_. Let the mean of *y*_*i*_ be $\bar {y} = \frac {1}{n} \sum _{i} y_{i}$, there will be the total sum of squares:

$${SS}_{\text{tot}} = \sum_{i} (y_{i} - \bar{y})^{2},$$

the regression sum of squares:

$${SS}_{\text{reg}} = \sum_{i} (f_{i} - \bar{y})^{2},$$

the residual sum of squares:

$${SS}_{\text{res}}=\sum_{i} (y_{i} - f_{i})^{2} = \sum_{i} e_{i}^{2},$$

*R*^2^ is defined as:

$$R^{2} = 1 - \frac{SS_{\text{res}}}{SS_{\text{tot}}} \,.$$

*R*_*p*_ measures the linear correlation between two variables. *Y* is used as the true label and *F* as the corresponding prediction for any sample. Let the mean and standard deviation of *Y* be $\bar {Y}$ and *σ*_*Y*_ respectively, and those for the prediction *F* be $\bar {F}$ and *σ*_*F*_ respectively. *R*_*p*_ is defined as:

$$R_{p} = \frac{\mathbb{E}}{\sigma_{Y}\sigma_{F}} \,.$$

RMSE measures the difference between two variables *Y* and *F*, and RMSE is defined as:

$$\text{RMSE} = \sqrt{\mathbb{E}} \,.$$

## Results

In this section, the performance of our model tCNNS is demonstrated under various data input settings. The titles and the meaning of these experiments are summarized as follows:

- Rediscovering Known Drug-Cell Line Responses. In this part, the drug-cell line interaction pairs are divided into a training set, a validation set and a testing set. tCNNS is trained on the training set and the result on the test set is reported. The validation set is used to decide when to stop training.
- Predicting Unknown Drug-Cell Line Responses. In this part, tCNNS is trained on the known drug-cell line interaction pairs in GDSC and is used to predict the missing pairs in GDSC.
- Retraining Without Extrapolated Activity Data. In this part, tCNNS is trained and tested on a subset of GDSC data. The subset is called max_conc data, and it is more accurate than the rest of the data in GDSC.
- Blind Test For Drugs And Cell lines. In this part, drugs and cell lines, instead of the interaction pairs, are divided into the training set, the validation set and the test set.
- Cell Lines Features Impacts. In this part, the performance of tCNNS is tested with respect to the different sizes of the feature vectors for the cell lines.
- Biological Meaning v.s Statistical Meaning. In this part, the input data are transformed in various ways to check whether tCNNS can capture the biological meaning in the data.
- Eliminating Outliers. The 223 drugs are visualized in different feature spaces to show that the features extracted from SMILES can solve the problem of outliers in traditional feature space.

### Rediscovering known drug-cell line responses

In the 223×948 (211,404) drug-cell line interaction pairs, GDSC provides the IC_50_ for 172,114 of them. To compare to the results of previous studies [2], the same procedure was employed. In this part, those known pairs were split into 80% as the training set, 10% as the validation set, and 10% as the testing set. This choice was made to guarantee any drug-cell line pair can only exist either in the training set or the test set. However, there was no restriction on the existence of drugs or cell lines. In each epoch, parameters in tCNNS were updated using gradient descent on the training set. The validation set was used to control the training of the tCNNS. If the RMSE on the validation set did not decrease in 10 recent epochs, the training process would stop and the predictions of our model on the testing set were compared with the given IC_50_ values in GDSC.

Experiments were set in this way to stimulate those real situations in which the models can only be trained on known interaction pairs between drugs and cell lines, and the models will be useful only if it can predict unknown interaction pairs. The validation set was separated from the training set so that it would be possible to choose a suitable time to stop training independently and avoid the problem of over-fitting.

**Fig. 2:**
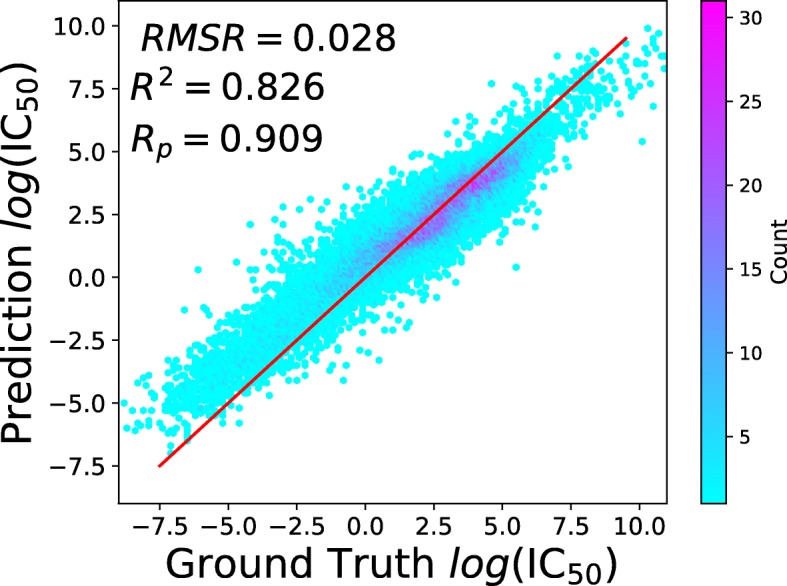
Regression results on the testing set compared to the ground truth IC_50_ values. The *x* axis is the experimental IC_50_ in natural logarithmic scale, and the *y* axis is the predicted IC_50_ in natural logarithmic scale. Different colors demonstrate how many testing samples fall in each small square of 0.1×0.1, or the hot map of the distribution, where dark purple indicates more samples (around 30 samples per small square 0.1×0.1) and light blue indicates fewer samples (less than 5 samples per small square 0.1×0.1)

tCNNS was tested 50 times, and an example of the regression result is displayed in Fig. 2.

In the 50 repeated experiments, *R*^2^ was increased from 0.72 to 0.826 for the mean and 0.831 for the top quartile. *R*_*p*_ was increased from 0.85 to 0.909 for the mean and 0.912 for the top quartile, and RMSE was reduced from 0.83 to about 0.027.

These results clearly showed that tCNNS outperformed the previous work reported in [2] in many ways, however, it should be pointed out that the comparison could be overly optimistic as the version of GDSC has changed so much and it is difficult to make a direct comparison. Instead, some indirect comparisons were made. After replacing the network reported in [2] with tCNNS, it did not converge using the features extracted from PaDEL. Then, the network in [2] was replaced with a deeper one, a network with three hidden layers and 1024 neurons in each hidden layer. This modified model got *R*^2^ of around 0.65 and *R*_*p*_ of around 0.81, which is shown in Additional file 1: Figure S1. It can be seen that the result was clearly horizontally stratified, which meant that the neural network lacked representational power using PaDEL features.

Many hyper-parameters affected the performance of tCNNS, such as the number of layers and the filter size. It was found that a smaller pooling size and more numbers of channels could further enhance the performance, but with a decrease in stability. For example, when the pooling size was reduced from 3 to 2, the top quartile *R*^2^ was further increased to 0.92 and the top quartile *R*_*p*_ was further increased to 0.96. The cost of this enhancement was that the network would become unstable and diverge [46] during the training. To keep experimental results repeatable, only the results with parameters that ensure stability are reported in this paper.

### Predicting unknown drug-cell line responses

**Fig. 3:**
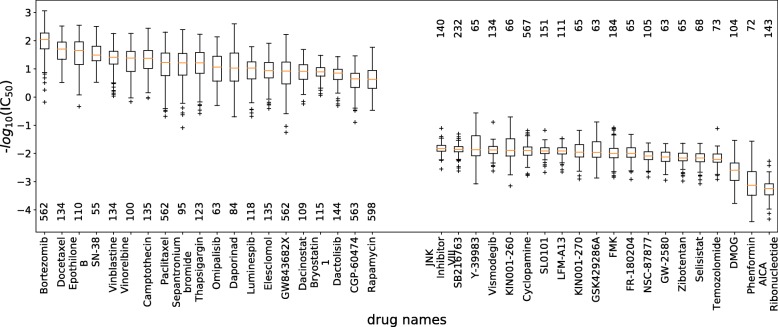
The predicted missing IC_50_ values. The drugs are ranged according to the median of their predicted IC_50_ values with cells. The horizontal axis denotes the drug names, and the vertical axis denotes their negative *log*_10_(IC_50_) values with cell lines. The left part is the top 20 drugs with lowest IC_50_ median, which means that they are probably the most effective drugs, and the right part is the last 20 drugs with the highest IC_50_ median, which means that they are the most ineffective drugs. For each drug, there is a number in its associated column, which is the number of cell lines whose interaction with the drug are missing in GDSC

In this part, tCNNS was trained on all the known interaction pairs in GDSC and then it was used to predict the values for those missing pairs in GDSC. The known pairs were split into 90% as the training set, and 10% as the validation set. Again, if the RMSE on the validation set did not decrease in 10 recent epochs, the training process would stop and the trained tCNNS was used to predict the values for the missing items. The results are shown in Fig. 3.

Figure 3 is the box plot of the predicted IC_50_ values for missing items grouped by drugs. For each drug, the box represents the distribution of the values with its related cell lines. Drugs were sorted by the median of the distribution: the 20 drugs with highest median and 20 drugs with the lowest median value were plotted. As the real values for these missing pairs were not known, the accuracy of our prediction was obtained by survey and analysis as follows.

*Bortezomib* was the best drug in our prediction. In fact, the top 40 pairs with the lowest IC_50_ value were all from *Bortezomib* with some other cell lines. The outstanding performance of *Bortesomib* in missing pairs was consistent with that in the existing pairs. There is some supporting information in [47] that the author found that drug *Bortezomib* can make cell lines to be sensitive to many other anti-cancer drugs.

*Aica ribonucleotide* and *Phenformin* have the poorest performance in tCNNS prediction. Based on our survey, the former one was initially invented to stop bleeding, and the later one was initially used as an anti-diabetic drug. These two drugs have the potential to cure cancer because they can inhibit the growth of cell (*Aica ribonucleotide*) or inhibit the growth of Complex I (*Phenformin*), but their effects are limited since anti-cancer is only the side effect of them, and not their main function.

Based on the tCNNS predictions, the IC_50_ of drug *Bortezomib* with cell line *NCI-H2342* was 1.19∗10^−4^*μg*. The small value indicated that there may be a good therapeutic effect. This prediction was supported by the findings reported in [48, 49], in which it is highlighted that *Bortezomib* is able to control Phosphorylation that causes lung cancer and *NCI-H2342* is a lung cell line. Similar evidence to support this prediction can also be found in Cell Signaling Technology’s 2011 published curation set (https://www.phosphosite.org/siteAction.action?id=3131).

### Retraining without extrapolated activity data

For each drug in GDSC, there are two important thresholds called minimum screening concentration (min_conc), which is the minimum IC_50_ value verified by biological experiments, and maximum screening concentration (max_conc), which is the maximum IC_50_ value verified by biological experiments. In GDSC, any IC_50_ beyond these two thresholds is extrapolated, and not verified by experiments. In general, IC_50_ value within min_conc and max_conc are more accurate than those outside of the thresholds.

In the GDSC data that we used in this paper, only max_conc is provided, and there are 64,440 IC_50_ values below max_conc, which is about 37% of the whole existing 172,114 IC_50_ values.

**Fig. 4:**
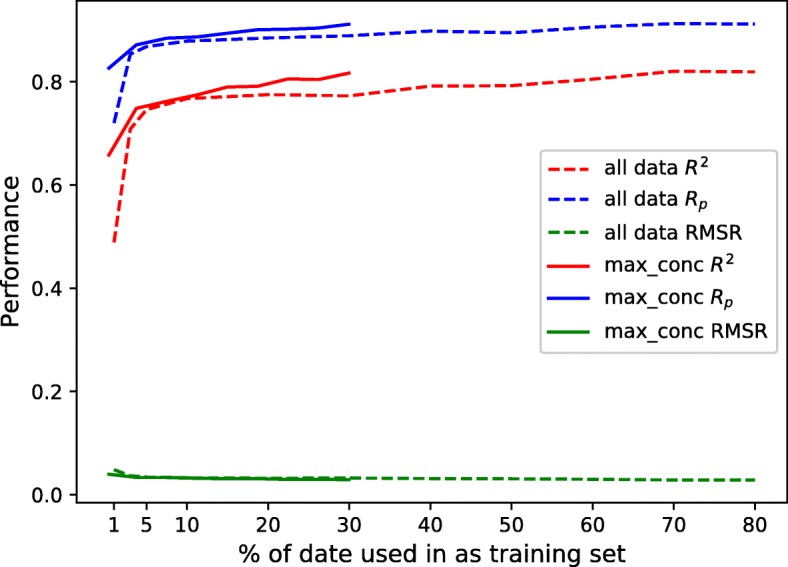
The performance with different percentages of data used. The x-axis is the percentage of data used as training data from the total existing IC_50_ values (172114) in the database. Since there is 10% for validating and 10% for testing, the max x is 80%. The y-axis is the top-quartile performance of our model. The solid lines represent the result on total existing data, and the dash lines represent the results where only the IC_50_ values below the max screening concentration threshold(max_conc) are used, below which the data is more accurate. Since there are only 64,440 values below max_conc, so the dash lines end at around $\frac {64,440}{172,114}*80\% = 30\%$

In this part, tCNNS was trained on the IC_50_ values below the max_conc threshold, which were randomly divided into 10% data for validating, 10% data for testing. The remaining 80% data is used for training and the size is reduced to 1% while the experiment was repeated 20 times. The regression result is shown in the Additional file 1: Figure S2. The comparison against the tCNNS which trained on whole existing data is shown in Fig. 4.

From Additional file 1: Figure S2, it can be observed that tCNNS can achieve almost the same good result just on max_conc data, which was faster because less data were needed. There were some other properties of tCNNS that could be concluded from Fig. 4. Firstly, it performed very well even with very limited training data. For example, when tCNNS was trained on only 1% of the existing IC_50_ values, *R*^2^ can be almost 0.5 and *R*_*p*_ be around 0.7. Secondly, and more importantly, tCNNS performed better with less and more accurate data. The dash lines (results on data below max _conc) were always above the solid lines (result on all data), and the final performance on max _conc data was almost as good as that on the total data, although the amount of data for the former was only 37% of the latter. To further compare the best performance on all data and max _conc data only, the distribution of the 20 times experiments are shown in Additional file 1: Figure S3.

There are three experimental results shown in Additional file 1: Figure S3, which are the experiments on all data, on the data below max _conc, and on a random subset of all data with the same size as those below max _conc. Comparing the result on data below max _conc with the result on the random data with the same size, it was observed that the performance of tCNNS was significantly better on data below max _conc than on random data with the same size, and it proved that tCNNS was able to utilize the information conveyed by accurate data.

### Blind test for drugs and cell lines

In previous experiments, interaction pairs between drugs and cell lines were randomly selected to be in the training set, the validation set, or the testing set, which meant that a specific drug or a specific cell line can exist in training and testing at the same time. This experimental setting corresponds to the problem of predicting the effect of a certain drug on a new cell line when its effect on another cell line is given. The problem becomes more challenging if the tested drug is a brand new one, and its effect on any cell lines is not known. To evaluate the performance of tCNNS on this challenging problem, a new experimental setting called *blind test* was designed.

In the blind test for drugs, drugs were constrained from existing in training and testing at the same time. The interaction pairs were divided based on drugs. 10% (23/223) drugs were randomly selected and their related IC_50_ values were kept for testing. For the remaining 90% drugs, 90% of their related IC_50_ values were randomly selected for training and 10% for validating.

In the blind test for cell lines, cell lines were prevented from existing in the training set and the testing set at the same time. The interaction pairs were divided based on cell lines. Similar to the case for drugs, 10% (94/948) cell lines were randomly selected and their related IC_50_ values were kept for testing. For the remaining 90% (904/948) cells, 90% of the related IC_50_ were used for training and 10% for validating.

**Fig. 5:**
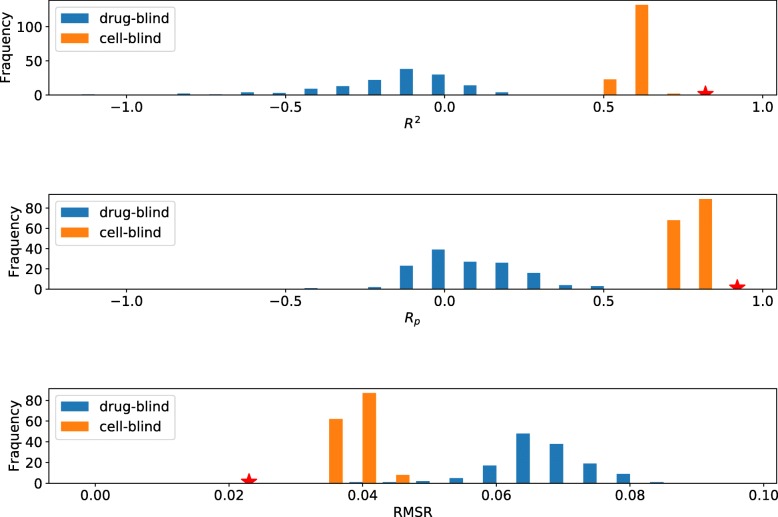
Drug and cell blind test result on total data. Yellow color boxes represent the result of cells blind, and blue color boxes for drugs blind. From top to bottom is the result for *R*^2^,*R*_*p*_ and RMSE respectively. The red star is the result without controlling data distribution

The blind test for drugs on all data and on the data below max _conc were repeated for 150 times respectively to check the distribution of the results. The same number of experiments for the cell lines were also conducted. The results on all data are shown in Fig. 5. The results on data below max_conc data are shown on Additional file 1: Figure S4 respectively.

From Fig. 5 and Additional file 1: Figure S4, it is observed that the performance of tCNNS was more robust with the blind test for cell lines but sensitive with the blind test for drugs. Without the knowledge of drugs in training, the performance dropped significantly. Comparing the results reported in Fig. 5 and in the Additional file 1: Figure S4, it can be observed that the extrapolated data made no contribution in this setting.

Comparing the results of the blind tests for drugs and for cell lines, the blind test for cell lines is slightly better, and the reason is that there is more common information shared among different cell lines and less among drugs. For example, cell lines share similar genetic information, but drugs can be very diversified. To reduce the information sharing among cells lines, another experimental setting was designed in which cell lines from the same tissue cannot exist in training and testing at the same time. The result was shown in Table 1.

**Table 1:** Tissue-Specific Test

Tissue name	Data amount	*R*^2^	*R*_*p*_	RMSE
Aero digestive	13806	0.703	0.843	0.0375
Tract		(0.826)	(0.916)	(0.0280)
Blood	31119	0.500	0.724	0.0449
		(0.833)	(0.917)	(0.0276)
Bone	6826	0.659	0.813	0.0405
		(0.825)	(0.915)	(0.0283)
Breast	9277	0.657	0.811	0.0383
		(0.829)	(0.919)	(0.0281)
Digestive	17200	0.667	0.817	0.0384
System		(0.830)	(0.918)	(0.0282)
Kidney	5199	0.669	0.819	0.0386
		(0.822)	(0.914)	(0.0286)
Lung	34086	0.614	0.784	0.0371
		(0.827)	(0.919)	(0.0285)
Nervous	15763	0.702	0.839	0.0364
System		(0.830)	(0.918)	(0.0280)
Pancreas	5358	0.703	0.840	0.0370
		(0.820)	(0.913)	(0.0287)
Skin	10488	0.676	0.824	0.0394
		(0.827)	(0.917)	(0.0281)
Soft tissue	3165	0.712	0.853	0.0384
		(0.821)	(0.914)	(0.0284)
Thyroid	2715	0.672	0.822	0.0410
		(0.833)	(0.918)	(0.0277)
Urogenital	17112	0.715	0.849	0.0363
System		(0.825)	(0.914)	(0.0282)

The first column is the 13 tissue names which are ranged in alphabetical order. The second column is the number of the ground true IC_50_ values for each tissue. The last three columns are *R*^2^, *R*_*p*_ and RMSE that our model tCNNS achieved by training on all the other tissue data. The number in the bracket is the result for the validation set

In GDSC, the 948 cell lines belong to 13 tissue types and 49 sub-tissue types. The 13 tissue types were used instead of 49 sub-tissue types because it can increase the distances and reduce the similarities among different tissues. Each time one tissue type was selected as testing data. For the rest of the tissues, they were mixed together and split into 90% for training and 10% for validation. From Table 1, it can be seen that the performance decrease differently for different tissues. For example, blood has the lowest *R*^2^ and *R*_*p*_ in all tissues, which indicated that blood is the most different tissue from other tissues.

### Cell lines features impacts

**Fig. 6:**
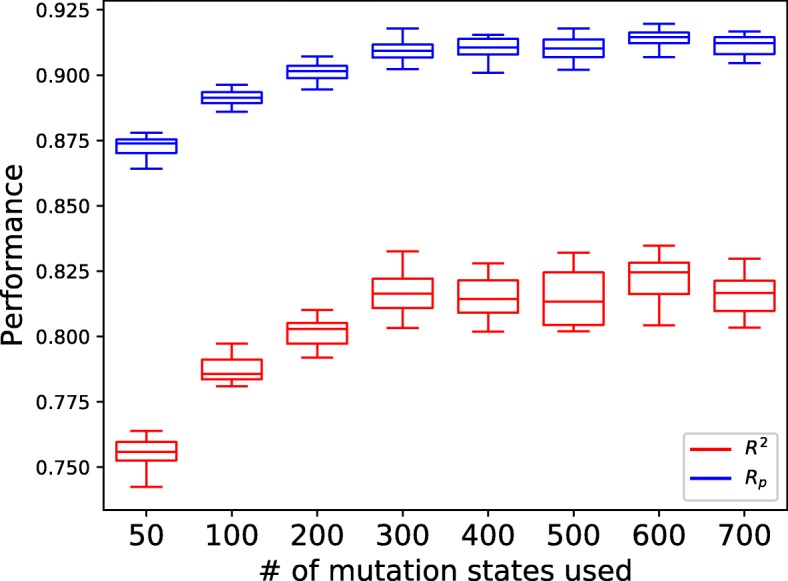
Sensitivity to the number of features. The x-axis is the number of mutation states used for cells in the experiments, and the y-axis is the performance achieved by tCNNS

In GDSC, the 735 features for cell lines after preprocessing belongs to 310 gene mutation states, and 425 copy number variations. As different laboratories may use different methods to extract the features for cell lines, in reality, it is not easy to have the complete 735 features for all cells. Besides, researchers may also have smaller and different feature groups for cell lines. It is attractive if tCNNS can have good performance with fewer features for cell lines. In this part, tCNNS performance was tested with different smaller numbers of features for cell lines to check the change of the performance with respect to the change of numbers of features for cell lines. The corresponding results in this part are shown in Fig. 6.

### Biological meaning v.s statistical meaning

tCNNS takes the one-hot representation of the SMILES format as the features for drugs. Initially, in the one-hot representation of the SMILES format, each row represents a symbol, and each column represents a position in the SMILES format, which is left aligned. For researchers, the SMILES format is a well-defined concept with biological meaning. However, tCNNS may lack the ability to comprehend the biological meaning of the SMILES format and it instead relies on the statistical pattern inside the data. To verify this hypothesis, the one-hot representation of the SMILES format was modified in three ways as follows:

1. The order of the symbols was randomly shuffled, which equals shuffling the rows in the one-hot representation.
2. The SMILES format was cut into two pieces, and the positions of which were switched. It is equivalent to shift the columns in the one-hot representation.
3. The positions in the SMILES format were shuffled, which equals to shuffling the columns in the one-hot representation.

The experiments were repeated 10 times in the three settings respectively and the results were compared with those obtained by using the SMILES format without any modification. The comparison is shown in Additional file 1: Figure S5. In the last two ways of the modification, the biological meaning of SMILES is corrupted. Initially, it was expected that the only the result of the first modification would be the same with the benchmark. It was surprising to see that the performances were similar in all three modifications. The stability among these results mean that tCNNS actually does not capture the biological meaning of the SMILES format for drugs, and it relied on the statistical patterns inside the SMILES format, cell line features, and the IC_50_ values.

### Eliminating outliers

In the last column of the Additional file 1: Figure S5, the results of tCNNS are compared with that of the baseline work [2]. As GDSC has been changed in recent years, it was impossible to use the same data as [2]. In the experiment, the method introduced in [2] was applied to current data. PaDEL(version 2.1.1) was used to extract 778 features for each drug. For cell lines, 735 features were used, instead of the 157 features used in the old version of GDSC [2].

To check the differences between the features extracted using PaDEL and the features extracted from the SMILES descriptions using CNN, the distribution of the drugs were visualized in different feature spaces. In a deep neural network, the fully connected layer is responsible for regression analysis, and CNN is used for extracting high-level features from the drug features. The input data for the fully connected network is the output of CNN tranche. Hence when drawing the distribution of drugs using CNN, the output of the last layer of CNN tranche was used for drugs.

**Fig. 7:**
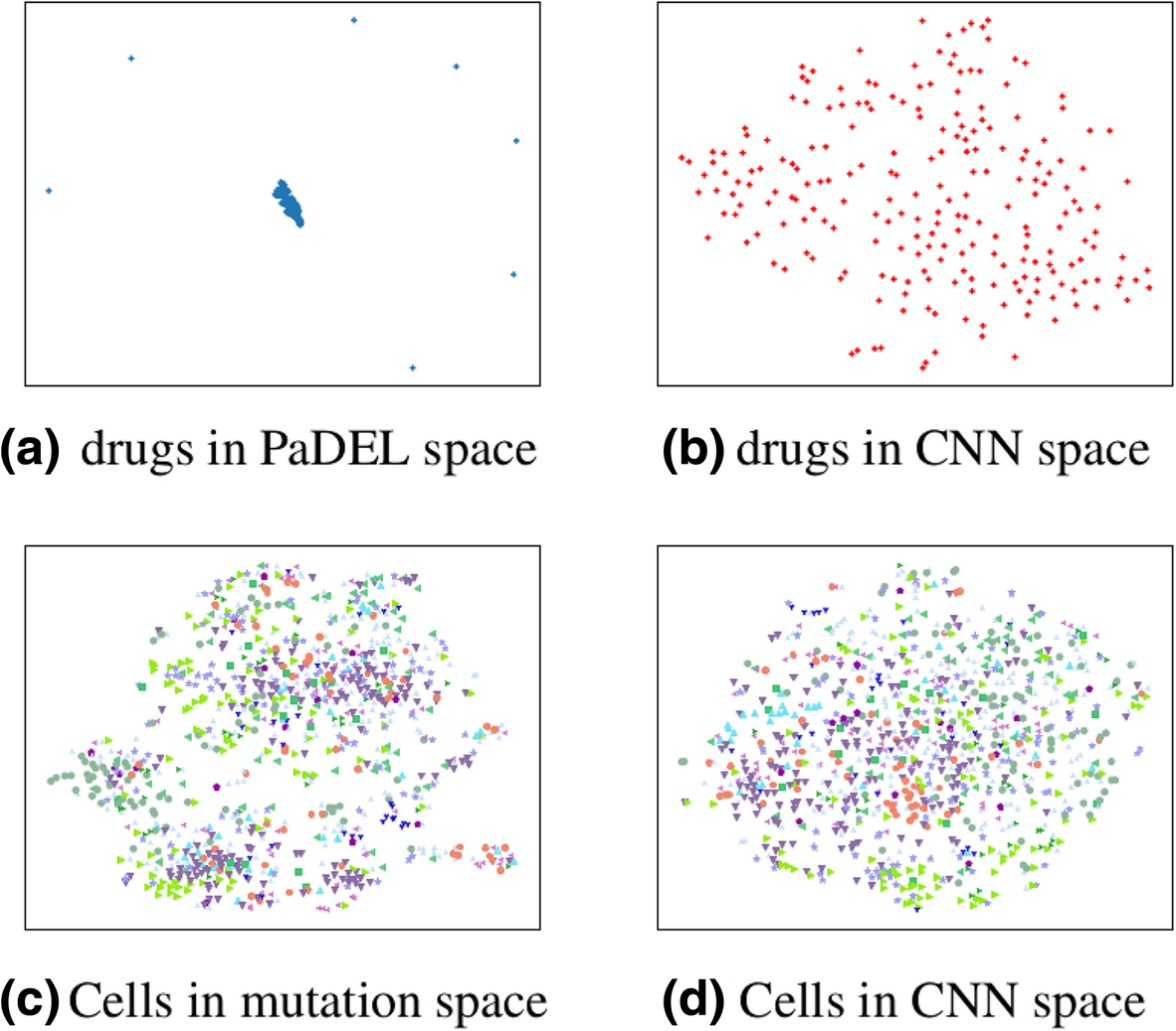
Visualization of drugs and cells in high-dimensional space. **a**) Drugs in PaDEL space (778 dims), **b**) Drugs in CNN space (420 dims), **c**) cells in mutation space (735 dims), and **d**) cells in CNN space (1680 dims)

The distribution of cell lines in genetic features space of GDSC was also compared with that found in the output space of the last layer in CNN. The visualization tool used was t-SNE [50], which was widely used to visualize high dimensional data in deep learning. The visualization results are shown in Fig. 7. It can be seen that there were 7 outliers in the PaDEL space for drugs. However, the problem does not exist in the features space for drugs extracted by tCNNS.

To conclude, there are seven subsections in this section, and they are summarized as follows:

- Rediscovering Known Drug-Cell Line Responses. In this part, tCNNS was trained on 80% data as the training set and tested on the other 10% data. The remaining 10% data were used as a validation set to decide when to stop training. The experiment was repeated 50 times and tCNNS achieves 0.826, 0.909 for mean *R*^2^, *R*_*p*_ respectively, and 0.831, 0.912 for top quartile of *R*^2^, *R*_*p*_ respectively.
- Predicting Unknown Drug-Cell Line Responses. tCNNS was used to predict the missing interaction pairs in GDSC. A literature survey was carried out and some published works that support the predictions of tCNNS were found and discussed.
- Retraining Without Extrapolated Activity Data. tCNNS was trained on max_conc data. Those IC_50_ values below max_conc are divided as 80% for training, 10% for validation and 10% for testing. Figure 4 shows that no statistically significant difference can be found between the results of tCNNS trained on data below max_conc and that on the whole data in 4.1.
- Blind Test For Drugs And Cell lines. Drugs and cell lines were restricted from existing in the training set and the testing set at the same time. In the blind test, the performance of tCNNS drops significantly, especially in the drug blind test where the mean of *R*_*p*_ drops to 0.2 and the mean of *R*^2^ drops to barely above 0.
- Cell Lines Features Impacts. The number of features for cell lines was reduced from about 700 to about 50. The mean of *R*^2^ remains above 0.80 when the number of features drops to 300, and it is still above 0.72 even when the number of features drops to 50.
- Biological Meaning v.s Statistical Meaning. As the results remain almost the same with different modifications to the input data, it can be concluded that tCNNS relies on the statistical pattern, instead of capturing the biological meaning in the data.
- Eliminating Outliers. 7 outliers exist in the traditional drug feature space. However, this problem does not exist in the feature space extracted from SMILES by tCNNS.

## Discussion

In Fig. 2, it can be observed that tCNNS is most accurate in the middle part, but less accurate in the two ends in the figure. In the bottom left corner, the input IC_50_ values are small but the predictions of tCNNS are incorrectly large. In the top right corner, the input IC_50_ values are large but the predictions of tCNNS are incorrectly small. This means that tCNNS can be further optimized if it can enhance its performance in these two areas.

Based on these results, it is concluded that the connections between the IC_50_ values and the SMILES format of drugs are stronger than those observed between the IC_50_ values and the features extracted using PaDEL.

Although the extrapolated data cannot enhance the accuracy of the model, they can help to improve scalability. The tCNNS model trained on all data performs well when tested on data below max_conc, which is natural because the later is a subset of the former. However, the model trained on data below max_conc performs poorly when tested on all data. *R*^2^ drops to 0.33 and *R*_*p*_ drops to 0.6. When tCNNS is trained on all data, it learns general knowledge which is useful on the whole dataset. That is why its performance remains stable when tested on data below max_conc. On the other hand, when tCNNS is trained on data below max_conc, it learns knowledge that only be applicable to this specific subset of all data. Its performance is dragged down by data above the max_conc when tested on all data. Although the performance of tCNNS on all data and data below max_conc is similar, the paths they achieve the performance are different.

Comparing the result on data below max _conc with that on all data, it can be seen that the means of *R*^2^ were almost the same, and the mean of *R*_*p*_ on all data was only a bit better than that on the data below mac _conc. Moreover, the variations of *R*^2^ and *R*_*p*_ on data below max _conc was a bit bigger than those on all data. To conclude, the contribution from low quality extrapolated data was limited, and they can only reduce variation and improve *R*_*p*_ a bit.

The results in the blind test give us some hint that with limited budgets, the in vivo experiment should be carefully arranged to cover a wider range of drugs and cells from different tissues to get better *in silico* predicting power.

It is very important to have the specific drug or cell line in the training stage before the performance is predicted. Experimental results support that even with only one or two related IC_50_ value, the performance will be significantly improved. For example, *NCI-H378* is a special cell line for lung cancer in GDSC, and there are only two IC_50_ values records for it. For other cell lines, all of them have at least 20 IC_50_ values. tCNNS can still make accurate predictions one of the IC_50_ values for *NCI-H378* if the other value is used during the training. Based on the results, the best drug for *NCI-H378* is *Bortezomib*, which has been previously recalled [48, 49].

Moreover, tCNNS predicts another potential drug *Docetaxel* for them. The predicted IC_50_ value between *Docetaxel* and *NCI-H378* is 0.03*μg* (third smallest for *NCI-H378*), and the predicted IC_50_ between *Docetaxel* and *NCI-H250* is 0.04*μg* (forth smallest for *NCI-H250*). It is reported in [51] that *APR-246* is a potential useful drug on lung cancer because of its synergy with *TP*53 mutations in lung cells, and there is an *"additive effects"* between *APR-246* and *Docetaxel*.

The visualization result of outliers highlights that CNN can distribute the drugs and cell lines more uniformly than features using PaDEL and features of GDSC. For drugs, those seven outliers in the PaDel space are exactly the seven drugs that are composed of multiple parts. The structures of the outliers are shown in the Additional file 1: Figure S6.

## Conclusion

In this paper, a model called tCNNS has been illustrated for phenotypic screening between cancer cell lines and anti-cancer drugs. tCNNS has been tested on a new version of GDSC with more data compared to previous works. It has achieved a much better coefficient of determinant and Pearson correlation than previous works and has made predictions for missing values in GDSC with trustful evidence. tCNNS can also converge with a very small set of training data and fewer features for cancer cell lines, which is economically efficient. tCNNS can take SMILES as input data for drugs, and this can solve the outlier problem occured in previous works where drug fingerprints are used as features.

## Additional file

Additional file 1 Supplementary. Some experiments results and figures are in the supplementary file of this paper. (PDF 378 kb)

## Data Availability

The authors declare that they have provided the code and data publicly accessible, which can be downloaded from https://github.com/Lowpassfilter/tCNNS-Project.

## Abbreviations

*R*^2^: Coefficient of determination
*R*_*p*_: Pearson correlation
RMSE: Root mean square error
tCNNS: Twin convolutional neural network for drugs in SMILES format
