# Supplementary material for: Improving prediction of phenotypic drug response on cancer cell lines using deep convolutional network
Source: BMC Bioinformatics. 2019 Jul 29;20:408. doi: 10.1186/s12859-019-2910-6 (PMC6664725; doi:10.1186/s12859-019-2910-6)
Supplement: Supplementary file 1 — Supplementary. Some experiments results and figures are in the supplementary file of this paper. (PDF 378 kb) [file 12859_2019_2910_MOESM1_ESM.pdf]

Supplementary

Improving Prediction of Phenotypic Drug Response on Cancer Cell Lines Using Deep Convolutional Network

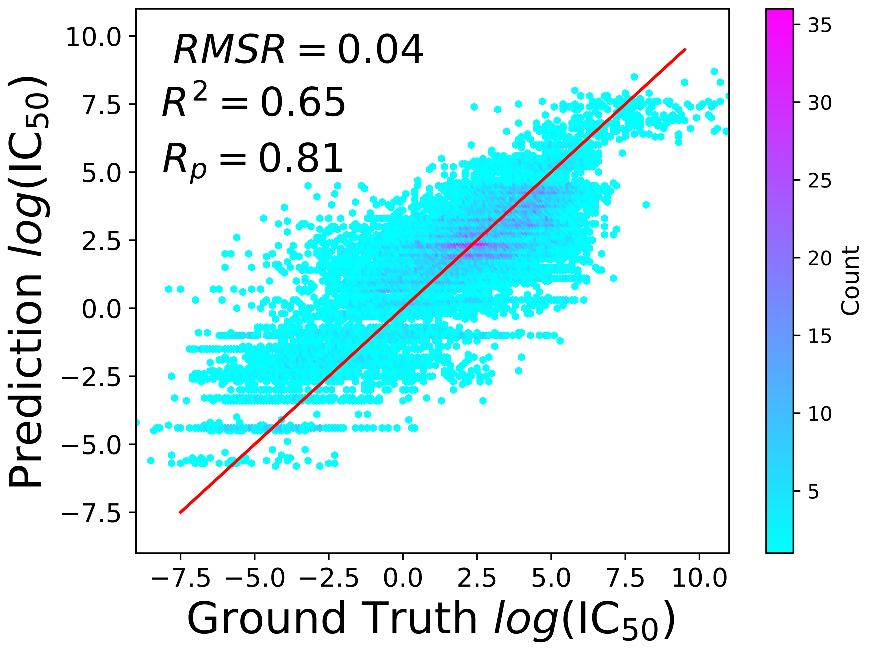

Figure 1: Regression result using PaDEL features for the drugs. We can see that the result is horizontally stratified, which indicates the lack of representation power for the network.

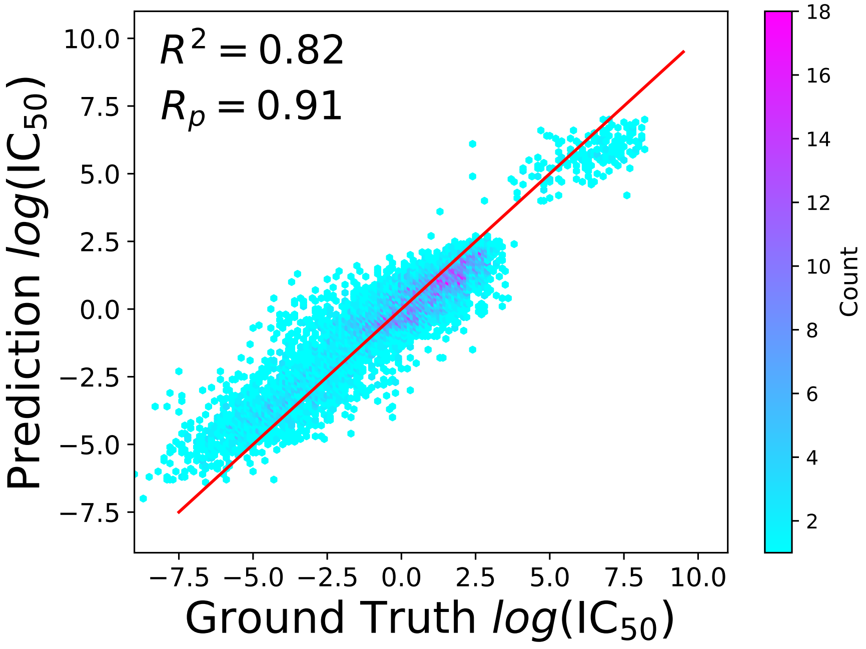

Figure 2: Regression result for max_conc data. R^2 and R_p are similar to the main result of the paper. The data below max_conc are not uniformly distributed, and they formed two distinguished groups.

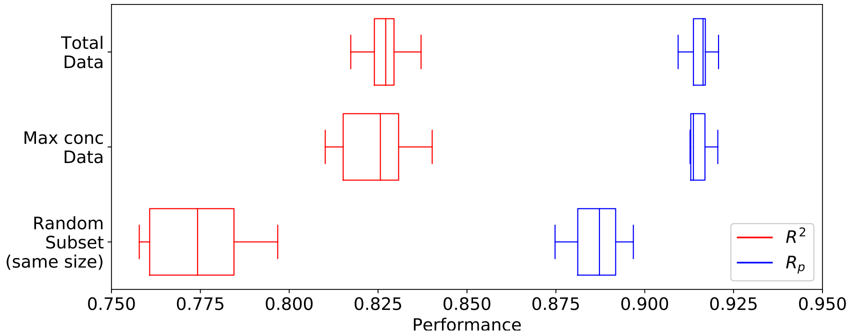

Figure 3: Influence of data quantity and quality. The first row is the result on all existing data ($172,114$ IC_{50} values), and the second row is the result on data below max_conc threshold ($64,440$ IC_{50} values), and the third row is the result on a random subset of all data with the same size as those below the max_conc threshold ($64,440$).

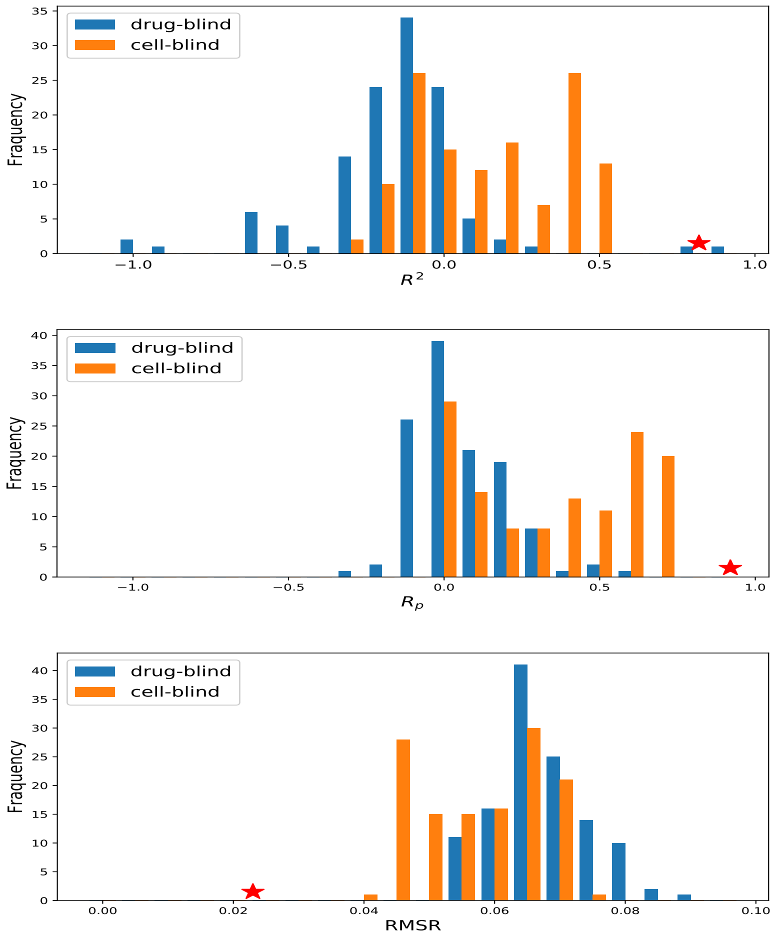

Figure 4: The blind test results for drugs and cell lines on data below the max_conc threshold. The yellow color boxes represent the result of cell lines, and the blue color boxes represent the result for drugs. From up to down is the result for R^2, R_p and $RMSR$ respectively. The red stars are the results in normal experiments.

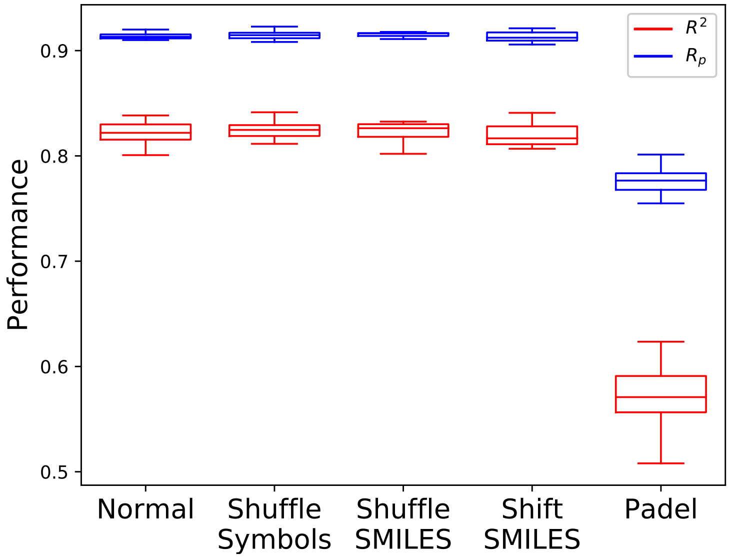

Figure 5: Performance of different ways to modify the input data. The first column is the original setting we use. The second column is the result by shuffle the order of symbols, the third column is the result by shuffle the SMILES, the fourth column is the result by shifting the SMILES. The last column is the result using the PaDEL features for drugs, just like the setting in the benchmark.

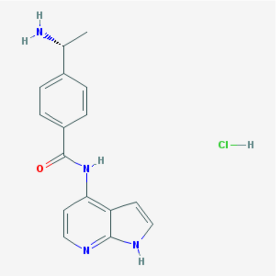

Y-39983

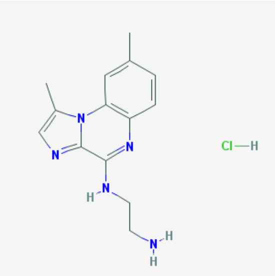

BMS-345541

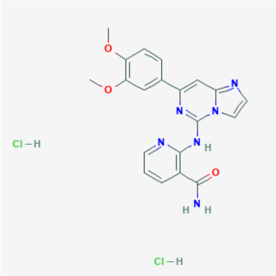

BAY-61-3606

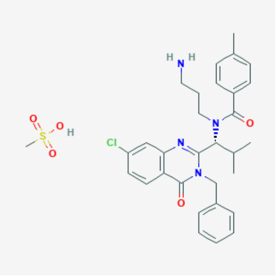

Ispinesib Mesylate

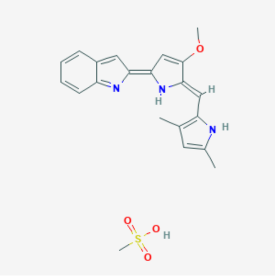

Obatoclax Mesylate

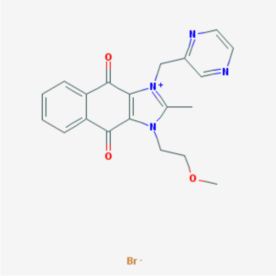

Sepantronium bromide

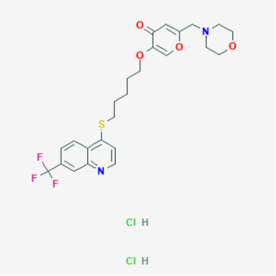

EHT-1864

Figure 6: The seven outliers in PaDEL's feature space for drugs.
